# Pilot Study to Improve In Vitro Identification of Culprit Drugs in SJS/TEN Using Granzyme B and Granulysin Secretion Assays

**DOI:** 10.1155/jimr/8497493

**Published:** 2026-04-10

**Authors:** Yuttana Srinoulprasert, Somkiat Ud-naen, Tunsuda Tansit, Papapit Tuchinda, Chamard Wongsa, Jettanong Klaewsongkram

**Affiliations:** ^1^ Department of Immunology, Faculty of Medicine Siriraj Hospital, Mahidol University, Bangkok, Thailand, mahidol.ac.th; ^2^ Department of Dermatology, Faculty of Medicine Siriraj Hospital, Mahidol University, Bangkok, Thailand, mahidol.ac.th; ^3^ Division of Allergy and Immunology, Department of Medicine, Faculty of Medicine Siriraj Hospital, Mahidol University, Bangkok, Thailand, mahidol.ac.th; ^4^ Division of Allergy and Clinical Immunology, Center of Excellence for Skin and Allergy Research, Faculty of Medicine, Chulalongkorn University, Bangkok, Thailand, chula.ac.th

**Keywords:** granulysin, granzyme B, lymphocyte transformation test, Stevens–Johnson syndrome (SJS)/toxic epidermal necrolysis (TEN)

## Abstract

**Introduction:**

Stevens–Johnson syndrome (SJS)/toxic epidermal necrolysis (TEN) are severe cutaneous adverse drug reactions (SCARs) with high morbidity and mortality. Accurate identification of the culprit drug is critical and remains challenging, whereas current in vitro tests such as lymphocyte transformation test (LTT) have limited sensitivity. To evaluate measuring granulysin (Gr) and granzyme B (GZB) secretion from drug‐stimulated peripheral blood mononuclear cells (PBMCs) for identification of culprit drugs and to compare their diagnostic performance with conventional LTT.

**Materials and Methods:**

PBMCs from 12 patients with confirmed SJS/TEN were incubated with suspected drugs. LTT and Gr/GZB detection assays were performed on day 3 and 6. Levels of Gr and GZB were quantified from supernatants, and results were normalized as production ratios. Cut‐off values were defined using receiver operating characteristic (ROC) curve analysis with high specificity thresholds.

**Results:**

GZB detection on day 3 yielded the highest sensitivity (8/12) compared to LTT (6/12) and Gr detection. Combining GZB and Gr detection improved the positive rate to 10/12. ROC curve analysis demonstrated higher AUCs for cytokine assay than for LTT, particularly for GZB at day 3. Combining GZB and Gr detection with LTT did not significantly increase diagnostic yield.

**Conclusion:**

The detection of GZB and Gr production in PBMC cultures provided a more sensitive and specific method than the conventional LTT for identifying culprit drugs in SJS/TEN. In particular, GZB detection at day 3 provided a shorter assay duration, avoided radioactive labeling, and demonstrated superior diagnostic performance. This cytokine‐based approach may represent a practical and safer alternative to enhance drug causality assessment in patients with SCARs.

## 1. Introduction

Adverse drug reactions (ADRs) are a significant global public health problem [1]. According to the World Health Organization, ADR is a body response that cannot be expected or intended to occur to drugs normally used for treatment [2]. ADR can be broadly categorized based on pharmacological mechanism type A (predictable, dose‐dependent) and type B (unpredictable, dose‐independent) reactions [2, 3]. Type B reactions, which include immune‐mediated responses, can cause a variety of symptoms from mild skin rash to a more severe skin injury called severe cutaneous ADRs (SCARs) [4–6]. Among these symptoms, Stevens–Johnson syndrome (SJS)/toxic epidermal necrolysis (TEN) is a rare but devastating T cell‐mediated hypersensitivity reaction characterized by majority of epidermal necrosis and detachment. SJS has a mortality rate ranging from 5%–15%, while TEN, the more extensive form, can reach mortality rates as high as 50% [7, 8].

Early and accurate identification of the culprit drug is important for patient safety and prevention of reexposure, yet culprit drug identification remains challenging due to the complexity of patient comorbidities with combination drugs used for concurrent treatment and limitations of available diagnostic methods [9, 10]. In vivo provocation testing is considered the gold standard for confirming drug causality, but it carries unacceptable risks in SJS/TEN due to the potential to trigger life‐threatening reactions. In vitro assays, such as the lymphocyte transformation test (LTT), provide a safer alternative by measuring proliferation of drug‐specific T cells upon reexposure to the suspect drug [11]. However, the LTT has low sensitivity in identifying culprit drugs in case of SJS/TEN, likely due to immunoregulatory factors in the recovery phase and the cytotoxic nature of the response itself [12, 13].

Recently, advances in our understanding of the immunopathogenesis of SJS/TEN have highlighted the pivotal role of cytotoxic T lymphocytes (CTLs), particularly the secretion of granulysin (Gr) and granzyme B (GZB), as key mediators of skin injury in affected patients [14–16]. These molecules are not only elevated in serum and blister fluid during acute disease but also reflect drug‐specific immune activation. Despite their mechanistic relevance, limited studies have evaluated their utility in in vitro diagnostic assays for culprit drug identification during the remission phase. Porebski et al. [17] demonstrated that cytokine secretion assays, including Gr and GZB, could enhance sensitivity beyond that of conventional LTT in SJS/TEN cases.

At present, standardized assays integrating these mediators into routine in vitro diagnosis of drug allergy remain underexplored. Therefore, we aimed to determine whether measuring Gr and GZB production in supernatants of peripheral blood mononuclear cells (PBMCs) stimulated with suspected drugs could enhance sensitivity for culprit drug identification in SJS/TEN. We also compared the performance of this cytokine detection assay with standard LTT, using receiver operating characteristic (ROC) analysis to evaluate diagnostic accuracy. Our study proposes a novel yet practical in vitro approach to prioritize a safer and potentially more accurate diagnostic alternative, aligning with recent efforts to optimize in vitro testing for severe drug hypersensitivity.

## 2. Materials and Methods

### 2.1. Study Subjects

This study was designed as an exploratory pilot investigation due to the extreme rarity of SJS/TEN. Twelve patients previously diagnosed with SJS/TEN were prospectively enrolled between December 2021 and December 2024. Clinical criteria for the diagnosis of SJS/TEN was based on the standard clinical criteria of the RegiSCAR study, including sudden‐onset, painful, erythematous macules and atypical targets (Figures S1 and S2) following a flu‐like prodrome and a positive Nikolsky sign. Progressive lesions with widespread epidermal detachment and at least two mucosal sites involved were characterized. Body surface area of skin detachment was calculated to differentiate SJS from TEN [18–20]. Even diagnosis was confirmed by board‐certified dermatologists based on standard clinical criteria, only the case that was not completely given as SJS/TEN underwent skin biopsy and histopathologic examination to confirm full‐thickness epidermal necrolysis, shown in the supporting figures (Figures S3–S5). All donors were recruited according to ethical procedures and gave informed consent. Inclusion required that patients had fully recovered from severe cutaneous reactions, recovered from their acute reaction for at least 4 weeks but not more than 5 years, and had no signs of active inflammation (e.g., fever or clinical illness) at the time of blood collection. This study was approved by the Siriraj institutional review board, Faculty of Medicine, Siriraj Hospital, Mahidol University, Bangkok, Thailand (COA No: Si 198/2014).

### 2.2. PBMC Isolation and LTT

PBMCs were isolated from heparinized venous blood using Ficoll‐Paque PLUS (GE Healthcare Bio‐Sciences) followed by density gradient centrifugation. After washing and counting, PBMCs were resuspended in R9 culture media at a density of 2 × 10^6^ cells per milliliter (mL). LTT was carried out following a previously established protocol [11, 12]. Pure drug substances (acetaminophen, allopurinol, amoxicillin, carbamazepine, ceftriaxone, ethambutol, etoricoxib, ibuprofen, isoniazid, lamotrigine, levofloxacin, phenytoin, pyrazinamide, rifampicin, sulfamethoxazole, and trimethoprim) were purchased from Sigma Incorporation, USA. Other drugs (amikacin, clavulanate, metronidazole, phenobarbital, and tazobactam) were employed from clinically available ampule for intravascular administration. The pure substances were dissolved in the proper solvents and three distinct nontoxic concentrations were achieved by diluting them in RPMI‐1640. Each subject’s PBMCs were cultured for 6 days at 37°C with 5% CO_2_ in quintuplicate in 96‐well plates with the suspicious drugs (three concentrations). Diphtheria‐tetanus toxoid (dT) (Novartis) and phytohemagglutinin (PHA, Sigma Lifesciences) were used as positive stimulating controls, whereas R9 medium was utilized as a negative control. Cell proliferation was quantified using the ^3^H‐thymidine incorporation technique and expressed as counts per minute (cpm) on a beta counter. The stimulation index (SI) was calculated as the ratio of cpm from drug‐stimulated to unstimulated wells, with SI ≥2.0 considered positive [12, 13, 21].

### 2.3. Cytokine Detection

Given that CTLs typically release Gr and GZB 3–5 days following activation, culture supernatants were collected on days 3 and 6 from each of the five replicate wells before the addition of ^3^H‐thymidine on day 6 [22–25]. Samples were pooled and centrifuged twice at 1500 rpm for 10 min to remove debris and stored at −20°C until analysis. Clear supernatants were subjected to quantification for Gr and GZB by MILLIPLEX Human Immuno‐Oncology Checkpoint Protein Panel 2—Immuno‐Oncology, purchased from Merck. Multiplex cytokine detection was performed following the manufacturer’s instructions. The multiplex approach was selected primarily to maximize data recovery from rare patient samples and to provide a consistent platform for longitudinal kinetic analysis. To ensure sufficient analyte concentration for multiplex bead‐based quantification, 20 μL of supernatant was harvested from each of the quintuplicate culture wells and pooled into a single 100 μL sample per condition. This strategy was employed to maximize the signal‐to‐noise ratio for low‐abundance cytokines in rare SJS/TEN patient samples and to provide a sufficient volume for the dual‐time‐point (day 3 and day 6) kinetic analysis. To account for interindividual variability in basal cytokine levels, results were expressed as a production ratio (the cytokine level in drug‐stimulated wells divided by the level in medium‐only wells). There have not been determined generalized cut‐off point for Gr and GZB production used to identify culprit drug, ROC analysis and Youden index were used to suggest cut‐off point for the production ratio for each cytokine [12, 26]. The cut‐off points of production ratios were selected based on their ratios corresponding to the highest specificity outcome derived from ROC analysis (95%–100%).

### 2.4. Statistical Analysis

All analyses were performed using GraphPad Prism Version 10.6.1 (GraphPad Software, La Jolla, CA, USA). Area under curve (AUC) derived from the ROC analysis for LTT and cytokine release assays were compared following the principle of Hanley JA and MeNeil BJ [27, 28]. To minimize the risk of type I error associated with multiple comparisons across biomarkers and time points, Dunnett’s T3 test was applied to all AUC comparisons after the Brown–Forsythe and Welch ANOVA tests. A *p*‐value <0.05 was considered statistically significant.

## 3. Results

### 3.1. Donor Characteristic and Tested Drugs

Twelve volunteers were recruited from December 2021 to December 2024, as detailed in Table 1. The median age of the volunteer was 37. Four cases were male (33.3%) and eight patients were female (66.7%). The ALDEN and Naranjo Probability Scale scores for the suspected drug stimulants ranged from 4 to 6 and 1 to 7, respectively, according to the clinical causality assessment. The median time interval between the acute clinical event and the date of blood collection for in vitro investigation was ~12.5 months. PBMCs from individual volunteers might be incubated with more than one drug depending on clinical history of drug exposure. The tested drugs included 13 antibiotics and 6 nonantibiotic drugs.

**Table 1:** Characteristics of volunteers, assessment score, time interval and stimulant.

Case	Age	Sex	ALDEN score	Naranjo score	Time interval (subside to test)	Stimulants
SJS01	31	M	6	3	18 months	Allopurinol

SJS02	33	F	4	4	—	Amikacin

SJS03	22	F	4	4	9 months	Sulfamethoxazole
						Trimethoprim

SJS04	67	M	5	4	6.5 months	Allopurinol

SJS05	80	F	4	6	1 month	Isoniazid
						Pyrazinamide
						Rifampicin
						Ethambutol

SJS06	41	F	5	3	3.5 months	Sulfamethoxazole

SJS07	33	M	5	6	10 months	Lamotrigine
						Phenytoin
						Phenobarbital

SJS08	23	F	4	4	2 months	Amoxicillin
						Clavulanate
						Lamotrigine

SJS09	56	F	5	2	2 months	Metronidazole
						Ceftriaxone
						Tazobactam

SJS010	28	F	5	7	7 months	Carbamazepine

SJS011	46	F	4	4	9 months	Etoricoxib
						Levofloxacin

SJS012	88	M	4	1	4 years 6 months	Isoniazid
						Pyrazinamide
						Rifampicin
						Ethambutol
						Levofloxacin

### 3.2. Comparison of Efficacy Between LTT and Cytokine Detection Assays to Identify Culprit Drugs in SJS/TEN Patients

To assess drug‐specific T cell activation in SJS/TEN patients, we performed LTT and measured the release of hallmark cytokines, Gr and GZB, from PBMCs stimulated in vitro with suspected culprit drugs. Supernatants were collected on day 3 (D3) and day 6 (D6) for cytokine quantification. ROC analysis was employed to evaluate the diagnostic performance of LTT and cytokine detection assays, individually and in combination, as previously reported [12, 17, 26, 29]. ROC curves for LTT and cytokine detection assays were shown in Figure 1, with results expressed as the AUC, 95% confidence interval, and *p*‐value. GZB detection on both D3 and D6, as well as the combined Gr and GZB detection on the same day, provided high AUCs with statistically significant *p*‐values (<0.05) as illustrated in Figure 1c,e,g,h. These findings indicated a strong diagnostic potential for identifying culprit drugs in SJS/TEN patients. The AUC of conventional LTT used to identify culprit drugs in SJS/TEN was lower than that of the GZB detection assay on D3 and D6 (Figure 1b,c,e). The AUC of each method was compared with the AUC derived from LTT and GZB detection on D3, as demonstrated in Figure 1a. The analysis revealed that the efficacy of GZB detection on D3 was significantly superior to those of LTT, Gr detection, and the combined cytokine detection on D3. However, the efficacy of GZB detection on D3 was not superior to that of GZB detection on D6 and the combined detection of cytokine on D6. Therefore, these findings support the GZB detection assays as a more effective diagnostic tool than conventional LTT and Gr detection assay for identifying culprit drugs in SJS/TEN patients.

Figure 1Comparison of LTT and cytokine assays to identify culprit drugs in SJS/TEN patients. Cytokine levels (granulysin and granzyme B) measured in supernatants of PBMC cultures on day 3 and day 6 following in vitro stimulation with suspected culprit drugs. Data were derived from 12 SJS/TEN patients, utilizing supernatants pooled from quintuplicate wells for each drug condition. Receiver operator characteristic (ROC) curves were illustrated for (b) lymphocyte transformation test (LTT), (c) granzyme B (GZB) D3, (d) granulysin (Gr) D3, (e) GZB D6, (f) Gr D3, (g) combined GZB + Gr D3, and (h) combined GZB + Gr D6. The ability of each readout to distinguish culprit from nonculprit drugs was expressed by area under the ROC curve (AUC), 95% confidence interval (CI), and *p*‐value. The statistical significance of differences between LTT and other assays was determined by Brown–Forsythe and Welch ANOVA, followed by Dunnett’s T3 multiple‐comparison correction, with statistical significance at 0.05 shown in (a) tabular format.

**Figure:**
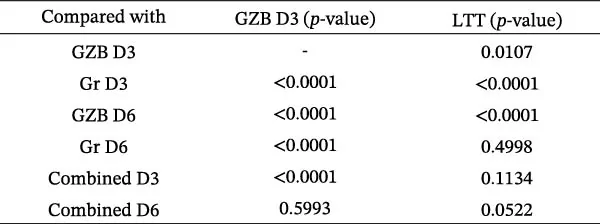
(a)

**Figure:**
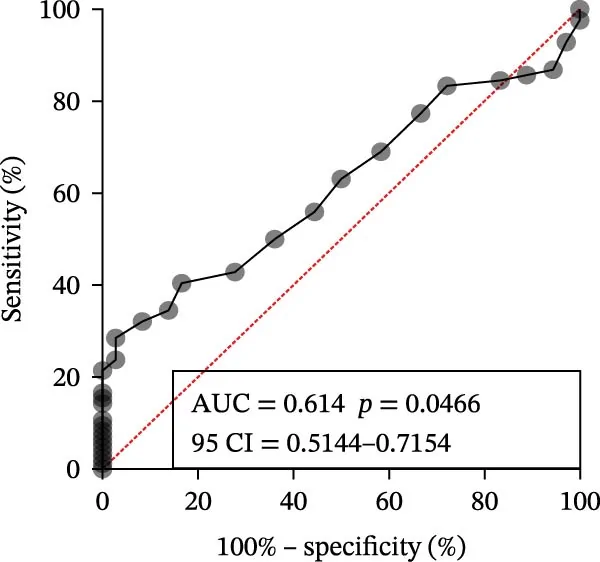
(b)

**Figure:**
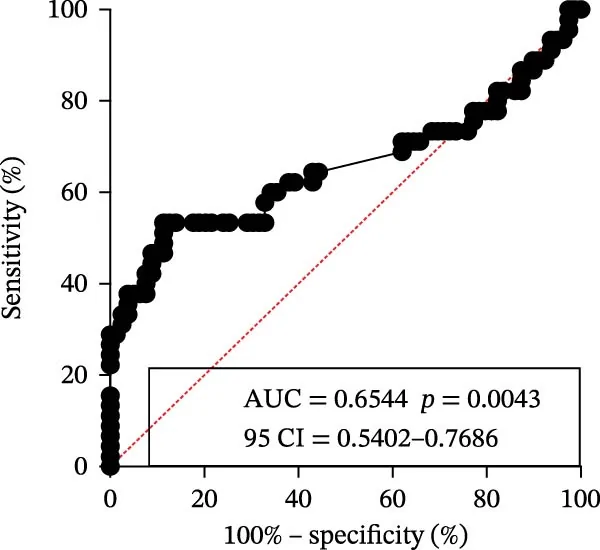
(c)

**Figure:**
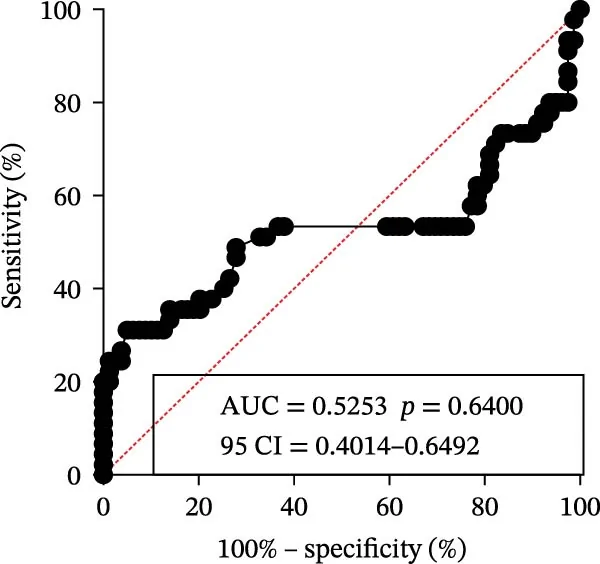
(d)

**Figure:**
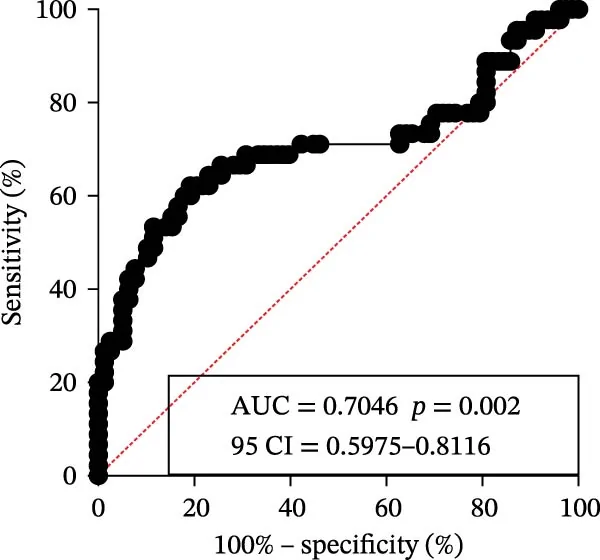
(e)

**Figure:**
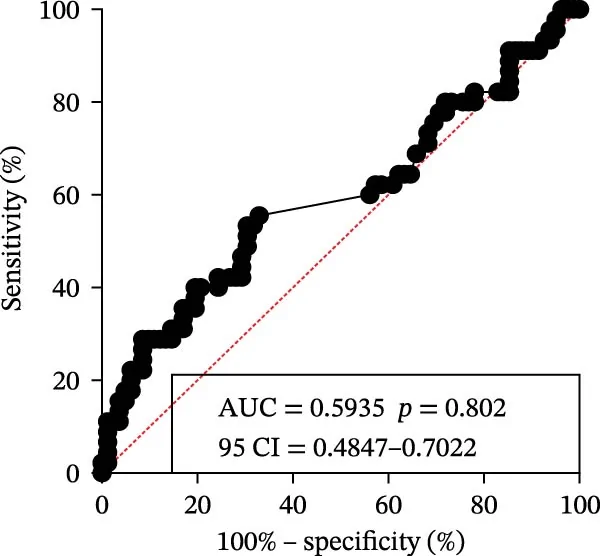
(f)

**Figure:**
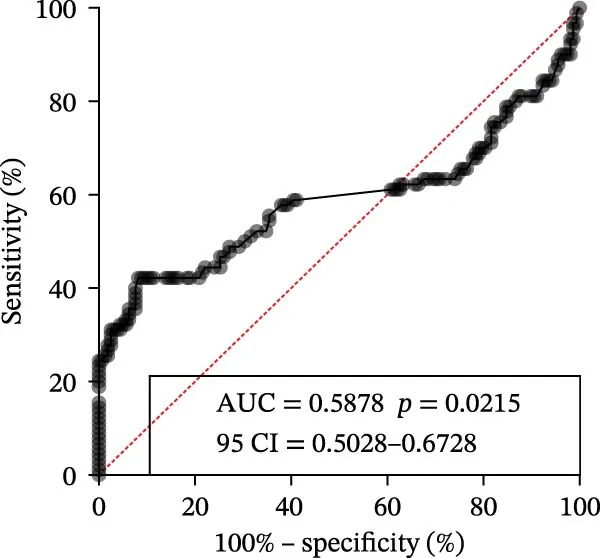
(g)

**Figure:**
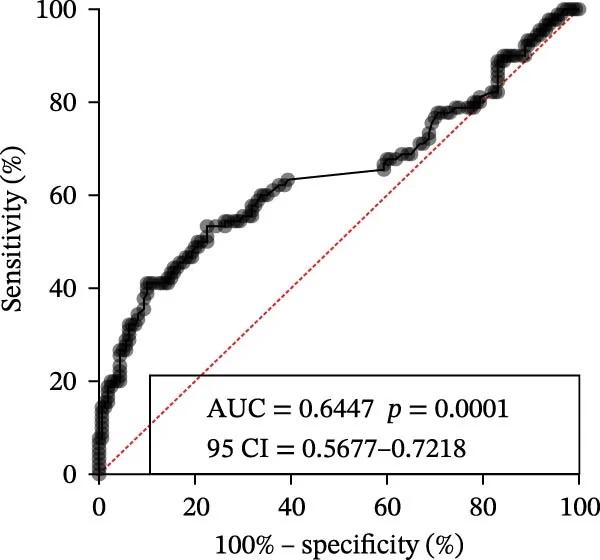
(h)

### 3.3. Evaluation of Diagnostic Performance of LTT and Cytokine‐LTT to Identify Culprit Drugs in SJS/TEN Patients

To evaluate whether the diagnostic sensitivity of the conventional could be enhanced by incorporating cytokine detection, we compared conventional LTT with its incorporation of GZB and Gr detection (cytokine‐LTT) to identify culprit drugs. ROC analysis revealed that the addition of any cytokine detection modestly improved AUC values (Figure 2c–h), although LTT alone yielded the lowest performance (Figure 2b). As shown in Figure 2a, the AUCs from cytokine‐LTT were significantly higher than those from LTT alone, particularly when GZB detection was included. Furthermore, combining GZB and Gr detection on D6 significantly enhanced the ability of LTT to identify culprit drugs.

Figure 2Enhanced detection of culprit drugs by LTT with cytokine detection assays. Diagnostic yield of conventional LTT versus LTT augmented with cytokine detection (granulysin or granzyme B) at D3 and D6 was demonstrated using data from 12 SJS/TEN patients (assays performed in quintuplicate). Receiver operator characteristic (ROC) curves were illustrated for (b) lymphocyte transformation test (LTT), (c) LTT + granzyme B (GZB) D3, (d) LTT + granulysin (Gr) D3, (e) LTT + GZB D6, (f) LTT + Gr D3, (g) LTT + combined GZB + Gr D3, and (h) LTT + combined GZB + Gr D6. The ability of each readout to distinguish culprit from nonculprit drugs was expressed by area under the ROC curve (AUC), 95% confidence interval (CI), and *p*‐value. The statistical significance of differences between LTT and other assays was determined by Brown–Forsythe and Welch ANOVA, followed by Dunnett’s T3 multiple‐comparison correction, with statistical significance at 0.05 shown in (a) tabular format.

**Figure:**
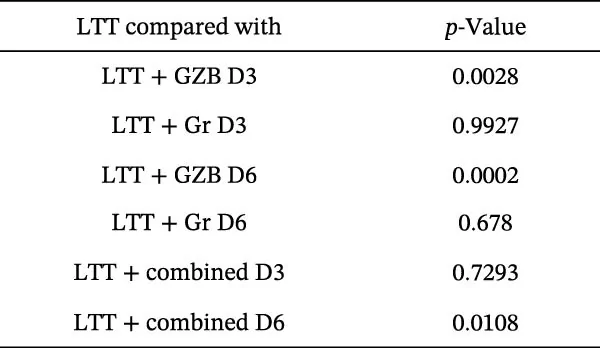
(a)

**Figure:**
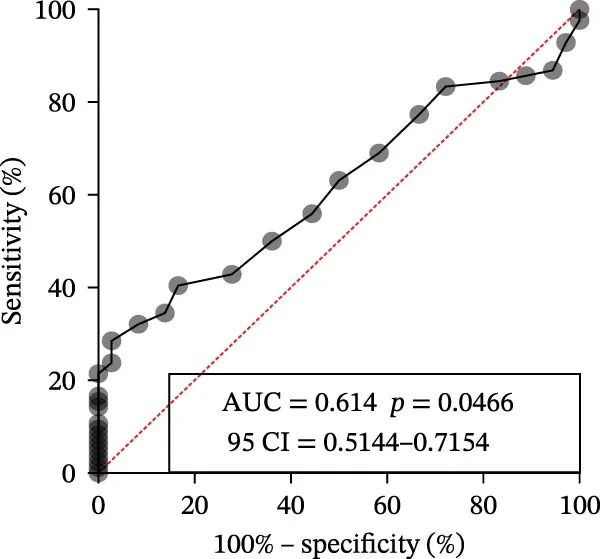
(b)

**Figure:**
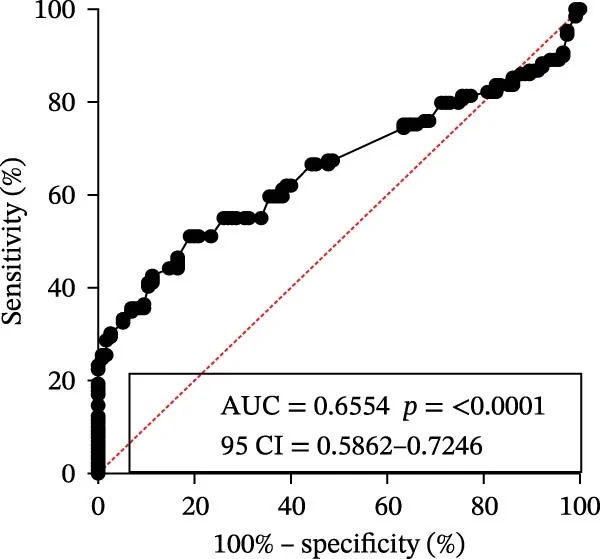
(c)

**Figure:**
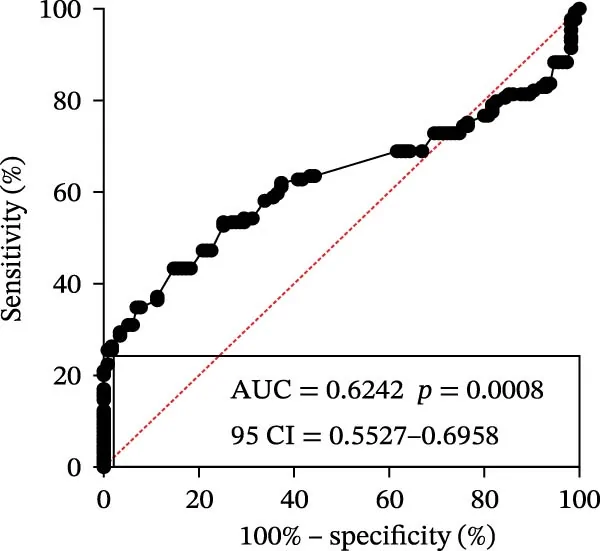
(d)

**Figure:**
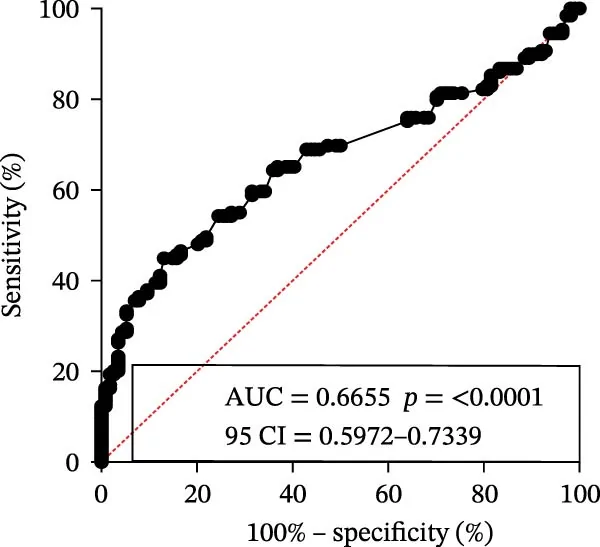
(e)

**Figure:**
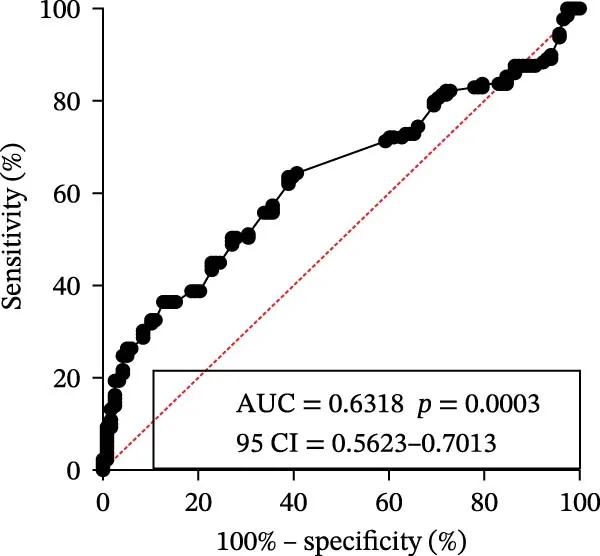
(f)

**Figure:**
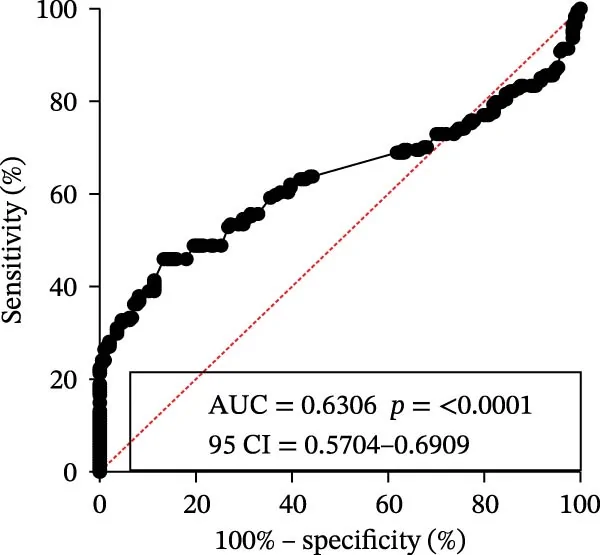
(g)

**Figure:**
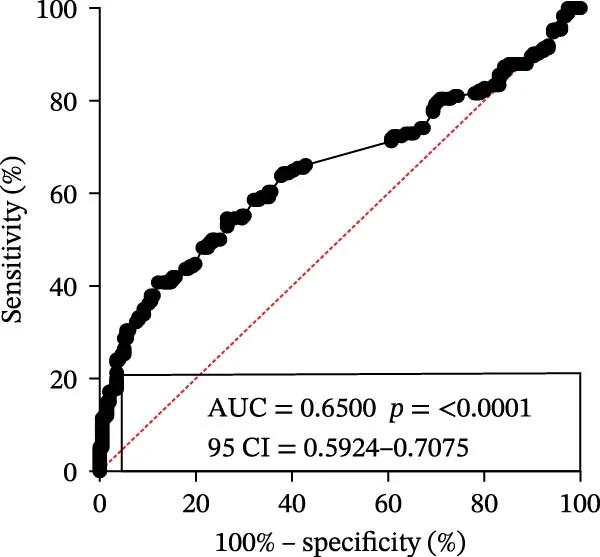
(h)

### 3.4. Comparison Sensitivity to Detect Culprit Drugs in SJS/TEN Using LTT, GZB, and Gr Production Assays

To evaluate the diagnostic performance of conventional LTT and cytokine detection assays, we applied cut‐off values of their readouts to define positive responses for each patient. The cut‐off value for the SI of greater than two was considered the standard threshold for a positive LTT result, as previously reported [12, 13, 21]. Although ROC analysis and the Youden index suggested appropriate cut‐off points, we selected cut‐off values corresponding to the highest specificity (almost 100%), prioritizing diagnostic accuracy over sensitivity. This approach aimed to match the specificity of cytokine assays to that of LTT, which typically ranges from 95 to 100% [30]. Based on this criterion, cut‐off values for Gr and GZB were set at 1.665 and 1.835 on D3 and 1.815 and 1.900 on D6, respectively. Therefore, the positive rates of identifying culprit drugs by LTT, Gr, and GZB detection on D3 and D6 were shown in Table 1 and Table S1.

LTT alone identified drug‐specific reactivity in 6 of 12 patients (50.0%), reflecting its limited sensitivity in the context of SJS/TEN. Cytokine readouts demonstrated that Gr detection was positive in 4/12 (33.33%) at D3 and 5/12 (41.67%) at D6, while GZB detection was positive in 8/12 (66.7%) at D3 and 6/12 (50.0%) at D6. Interestingly, these results revealed that the detection of GZB production on D3 provided a higher positive rate than that of LTT and Gr detection. When Gr and GZB detection on D3 were combined, the positive rate increased to 9/12 (75.0%), higher than the combined detection on D6 (6/12; 50.0%). When we incorporated GZB or Gr detection at D3 or D6 with LTT to identify culprit drugs, the positive rate increased from 50.0% to 83.33%. These findings suggested that incorporating functional cytokine detection into conventional proliferation assays significantly enhanced the ability of LTT to identify culprit drugs in SJS/TEN. Among the cytokine assays, GZB detection on D3 provided the highest sensitivity and appears to be a practical and informative time point for accurately identifying culprit drugs in patients recovering from SJS/TEN. Notably, none of the cytokine tests yielded positive results in the irrelevant drug control, supporting the high specificity of the assay (Table 2).

**Table 2:** Results of LTT, granzyme B and granulysin corresponding to tested drugs of volunteers.

Case	Stimulant	LTT (SI^b^)	Granzyme B^a^ D3	Granulysin^a^ D3	Granzyme B^a^ D6	Granulysin^a^ D6
SJS01	Allopurinol	1.6	**3.150**	0.377	0.883	1.238

SJS02	Amikacin	1.5	**3.270**	1.198	**4.978**	**5.385**

SJS03	Sulfamethoxazole	1.1	1.321	1.000	1.303	1.608
	Trimethoprim	1.4	1.789	**11.993**	**4.603**	**68.590**

SJS04	Allopurinol	1.4	1.800	1.258	1.415	1.316

SJS05	Isoniazid	**2.3**	1.573	1.044	**2.643**	**2.111**
	Pyrazinamide	**4.0**	0.617	0.755	**1.922**	1.285
	Rifampicin	1.9	1.194	0.984	**3.441**	**2.404**
	Ethambutol	**2.6**	1.442	1.122	**3.982**	**1.823**

SJS06	Sulfamethoxazole	**4.1**	**2.517**	**6.568**	0.981	0.751

SJS07	Lamotrigine	2.0	1.346	1.238	1.528	0.944
	Phenytoin	**7.4**	**3.213**	1.423	**20.625**	1.749
	Phenobarbital	1.2	1.096	1.125	1.751	1.101

SJS08	Amoxicillin	1.7	1.218	0.902	1.720	1.455
	Clavulanate	1.1	1.072	1.043	1.586	1.798
	Lamotrigine	**2.5**	**2.518**	**1.722**	1.521	1.340

SJS09	Metronidazole	0.5	**1.958**	**1.825**	0.721	0.954
	Ceftriaxone	0.4	1.083	0.968	0.800	0.796
	Tazobactam	0.5	**2.627**	**3.747**	1.280	0.921

SJS010	Carbamazepine	1.3	0.915	1.139	1.157	0.997

SJS011	Etoricoxib	0.6	1.632	1.608	1.037	0.898
	Levofloxacin	**9.6**	**13.439**	1.049	**16.119**	**3.368**

SJS012	Isoniazid	**2.8**	1.413	1.396	1.721	**4.073**
	Pyrazinamide	1.9	1.252	1.396	**2.525**	**5.736**
	Rifampicin	1.8	1.173	1.042	**2.455**	**3.105**
	Ethambutol	**2.1**	1.537	0.376	**2.105**	0.865
	Levofloxacin	**2.4**	**1.872**	0.499	**2.510**	1.293
	Positive rate (person)	6/12	8/12	4/12	6/12	5/12

*Note:* LTT results and cytokine (granzyme B and granulysin) productions derived from PBMCs of the patients reexposed with suspicious drugs in vitro assays. The cut‐off points on D3 for GZB and Gr production ratio were 1.835 and 1.665, respectively, as well as the cut‐off points on D6 for GZB and Gr production ratio were 1.900 and 1.815, respectively. Person who got positive result, when at least one suspicious drug was indicated as positive.

^a^The highest ratio of each cytokine with each individual’s baseline amount of the cytokine, positive results were expressed with bold when cytokine ratio was more than cut‐off points.

^b^The highest SI from three‐concentration induction, positive results were expressed with bold when SI > 2.0.

## 4. Discussion

In this study, we assessed the efficacy of cytokine detection assays that target GZB and Gr as alternative or adjunctive in vitro methods for identifying the causative drug in SJS/TEN patients. The selection of the MILLIPLEX multiplex assay was a strategic choice necessitated by the limited PBMC recovery and supernatant volumes typical of rare SJS/TEN samples. While conventional ELISA requires 4–5 times more volume per analyte, this high‐sensitivity platform enabled the simultaneous quantification of both Gr and GZB from a single 20 mL aliquot, ensuring sufficient stock for repeated validation if required. Furthermore, measuring all day 3 and day 6 samples under identical conditions within a single validated batch minimized interassay variability, a common complication in multiplate ELISA studies. This methodology provided a broad dynamic range essential for capturing low‐abundance cytotoxic signals across a diverse testing interval ranging from 1 to 54 months postreaction.

The cut‐off values in our study were equivalent to those reported by Chu et al. [31]. Compared to the conventional LTT, GZB detection at day 3 (D3) demonstrated superior sensitivity, identifying the causative drug in 8 out of 12 patients versus 6 of 12 for LTT. The highest overall positive rate (10/12) was achieved when combining GZB and Gr measurements at D3. However, combining LTT with cytokine assays did not improve sensitivity, suggesting that cytokine assays alone might be sufficient in many cases. GZB production at D3 retained high specificity when using optimized cut‐off values, making it a viable stand‐alone diagnostic tool.

Our finding of low sensitivity of LTT in SJS/TEN patients was also consistent with previous studies indicating limited sensitivity of LTT [13, 32]. It was likely due to the immunoregulatory mechanism in the convalescent phase or the inherent cytotoxic milieu of SJS/TEN [14, 16, 17]. Although Gr and GZB proposed as hallmark cytokines of SJS/TEN pathogenesis and may interfere with antigen‐specific T cell proliferation, cytokine detection assays have been proposed for use in identifying the causative drug [14, 33–35]. Porebski et al. [17] demonstrated that drug‐induced secretion of these molecules could reliably distinguish culprits from nonculprit drugs in affected patients. While our study and the work by Porebski et al. [17] emphasized the diagnostic value of GZB and Gr in drug hypersensitivity, we extended their findings by demonstrating that a 6‐day kinetic window was essential for providing diagnostic signal in SJS/TEN. Unlike the 72 h protocol in previous research, our results revealed that Gr concentrations frequently reached the higher concentration at day 6, reflecting the de novo synthesis and clonal expansion of pathogenic T cells that might be missed in shorter assays. Furthermore, by utilizing a multimarker approach, we identified a pathogenic signature of synchronized GZB and Gr increases that distinguished true culprit drugs from transient, nonspecific reactivity. This kinetic model offers superior sensitivity, particularly in cases where investigation occurs years after the acute event and precursor T cell frequencies are low.

A key strength of our study is the direct comparison of multiple assay formats (LTT, individual cytokines, and combined readouts) within the same patient samples, thereby minimizing intersample variability. We also prioritized specificity by selecting cut‐off values derived from ROC analysis that minimized false positives, maintaining diagnostic stringency. ROC‐derived thresholds (AUC and cutoff) were evaluated for internal stability with leave‐one‐out cross‐validation (LOOCV). The LOOCV revealed that mean AUC and sensitivity remained stable (<5% variance), suggesting that the findings were not significantly driven by overfitting within this specific cohort.

Although kinetic production of Gr and GZB was occasionally discordant with LTT outcomes, our data demonstrated that relying on a single time point or a single biomarker may lead to false negatives in identifying the culprit drug. The divergent kinetics of GZB and Gr observed in this study provided important insights into the underlying immunological mechanisms of SJS/TEN. The observation that some drugs elicited a rapid D3 response while others required 6 days of culture for a detectable signal suggested the different mode of cytotoxic mediator release by drug‐specific T cell responses. The D3 time point was highly sensitive for capturing rapid activations, whereas D6 was indispensable for detecting the de novo synthesis and pathogenic T cell clonal expansion. Assessing GZB on D3 has significant benefits for clinical practice: it reduces the diagnostic turnaround time by half compared to the 6‐day LTT and avoids the need for radioactive isotopes.

The incorporation of kinetic secretome monitoring into the diagnostic evaluation for SJS/TEN overcomes the inherent limitations of existing in vitro methodologies. By shifting the focus from how many cells are present (LTT) to how much pathogenic mediator is being produced (secretome kinetics). The practical advantages enable the incorporation of GZB assays into conventional clinical immunology workflows, particularly when LTT is inconclusive or logistically inaccessible. This function‐first approach is especially valuable in complex clinical scenarios involving polypharmacy or investigation for a remote history of allergic cases, ultimately enhancing patient safety and precision in SJS/TEN management. In light of the urgent need for safer and more precise in vitro diagnostics for SJS/TEN, our results supported the adoption of D3 GZB detection as a rapid primary screening tool for the definitive identification of culprit drugs. Moreover, the integration of both GZB and Gr across multiple timepoints yielded a more comprehensive and precise immunological signature, thereby enhancing the identification of the causative agents in SJS/TEN. While this study primarily focused on identifying culprit drugs, the excellent specificity of the assay could be significantly utilitized in the broader differential diagnosis of SJS/TEN. Although a negative result did not definitively rule out drug hypersensitivity, the absence of significant Gr and GZB secretion upon stimulation might serve as a significant indicator to consider other potential triggers of SJS/TEN, such as *Mycoplasma pneumoniae* or viral infections. Consequently, this assay serves as valuable adjunctive evidence to support the investigation of nondrug causes, thereby facilitating more accurate treatment planning and management strategies for patients where drug causality is ambiguous.

However, our study has limitations. (1) Due to the rarity of SJS/TEN, it was unachievable to recruit a cohort of significant size to fully validate the scope of this study within a short timeframe. Our study was instead a pilot study focused on prospectively enrolling all consenting patients who met the inclusion criteria at our two institutes over a 3‐year period. Therefore, the small sample size and heterogeneity of the culprit drugs limit generalizability. External validation in larger cohorts and across different drug classes is also necessary before broad clinical adoption. (2) Although pooling supernatants from quintuplicate wells prevented the evaluation of intraassay variability, this approach was necessary to ensure cytokine concentrations remained within the linear dynamic range of the MILLIPLEX platform, considering the limited PBMC recovery from rare SJS/TEN samples. Furthermore, as PBMCs across quintuplicate wells might not proliferate uniformly, pooling was employed to average these individual variations and yielded a more stable and representative sample for each experimental condition. Despite this limitation, the biological significance of our findings remained solid, as the significant kinetic changes—such as the sixfold increase in Gr between D3 and D6—greatly exceeded the usual 10%–15% technical variability associated with bead‐based assays. The repeated occurrence of unique kinetic characteristics across multiple independent drugs and patients indicated that these findings represented a substantial immunological phenomenon rather than mere technical noise. Further studies with larger blood volumes could improve these findings by providing a more detailed perspective on the specific cellular contributions to the cytotoxic secretome. (3) Drug‐specific memory T lymphocytes in SJS/TEN patients enabling causality assessment years or decades after the acute episode. The central (*T*_CM_) and effector (*T*_EM_) memory compartments contain these cells, which keep a pathogenic signature that can be reactivated in vitro. Strong Gr and GZB responses were detected even in cases with the greatest latency durations, confirming this persistence. A 6‐day culture period was used for clonal expansion to compensate for T cell precursor frequency decline. This duration effectively enriches and expands signals originating from limited initial populations of drug‐specific T cells. We used the SI to normalize the results so that we could make fair comparisons throughout different time periods and to demonstrate that the diagnostic threshold was consistent independent of the patient’s immunological status. Statistical analysis of SIs revealed that they were not significantly different between samples obtained within 12 months and those collected beyond 12 months. These data confirmed that SJS/TEN’s drug‐specific memory T cell repertoire persisted, detectable, and expandable for years after response. (4) Although we demonstrated that combination of detection for Gr and GZB improved sensitivity, the underlying mechanisms for differential cytokine kinetics and their correlation with disease severity or timing remain to be fully elucidated. Future studies should focus on multicenter validation, comparison with ELISpot or flow cytometry‐based assays, and adaptation for multiplex testing.

## Author Contributions

Yuttana Srinoulprasert conceived, designed the experiments, and analyzed data. Somkiat Ud‐naen and Tunsuda Tansit performed the experiments and analyzed data. Papapit Tuchinda, Chamard Wongsa, and Jettanong Klaewsongkram recruited patients. Yuttana Srinoulprasert wrote the manuscript.

## Funding

This research project was supported by the Faculty of Medicine Siriraj Hospital, Mahidol University (Grant [IO] R016333003). Papapit Tuchinda, Chamard Wongsa, and Yuttana Srinoulprasert were supported by the Chalermprakiat Fund.

## Disclosure

The funding sponsors had no role in the design of the study, the collection, analysis and interpretation of the data, the writing of the manuscript, or the decision to publish the results.

## Consent

The patients in this manuscript have given written informed consent to publication of their case details.

## Conflicts of Interest

The authors declare no conflicts of interest.

## Supporting Information

Additional supporting information can be found online in the Supporting Information section.

## Supporting information


**Supporting Information** Figure S1 illustrates the early clinical manifestation of Stevens–Johnson Syndrome (SJS), characterized by erythematous and dusky macules with irregular borders that tend to coalesce; certain lesions appear a “targetoid” appearance with central duskiness, indicating the onset of epidermal necrosis. Figure S2 represents an advanced presentation of the SJS/TEN overlap spectrum, exhibiting extensive, confluent purpuric macules and substantial regions of dusky, leaden‐hued erythema. The presence of flaccid bullae and noticeable epidermal detachment (positive Nikolsky sign) indicates extensive full‐thickness epidermal necrolysis, affecting a larger proportion of the total body surface area compared to Figure S1. Figure S3 presents a low‐magnification overview (X10) of the skin sample, illustrating a notable subepidermal cleft and significant detachment of the necrotic epidermis from the underlying dermis, characteristic of SJS/TEN. In Figure S4, higher magnification (X20) distinctly illustrates the full‐thickness epidermal necrolysis, characterized by a detached blister roof composed of eosinophilic, necrotic keratinocytes indicating a complete loss of cellular detail. Ultimately, the high‐power magnification in Figure S5 (X40) provides a comprehensive examination of the interface dermatitis, highlighting scattered dyskeratotic (apoptotic) keratinocytes, vacuolar degeneration of the basal layer, and a sparse perivascular lymphocytic infiltrate within the papillary dermis. Table S1: LTT results and cytokine (granzyme B and granulysin) productions derived from PBMCs of the patients reexposured with suspicious drugs in vitro assays.

## Data Availability

The data that support the findings of this study are available in the Supporting Information of this article.
