# Supplementary material for: Pilot Study to Improve In Vitro Identification of Culprit Drugs in SJS/TEN Using Granzyme B and Granulysin Secretion Assays
Source: J Immunol Res. 2026 Apr 10;2026:8497493. doi: 10.1155/jimr/8497493 (PMC13581063; doi:10.1155/jimr/8497493)
Supplement: Supplementary file 1 [file JIMR-2026-8497493-s001.docx]

Supplementary Figures

Figure S1	Figure S2

Figure S1 illustrates the early clinical manifestation of Stevens-Johnson Syndrome (SJS), characterized by erythematous and dusky macules with irregular borders that tend to coalesce; certain lesions appear a "targetoid" appearance with central duskiness, indicating the onset of epidermal necrosis. Figure S2 represents an advanced presentation of the SJS/TEN overlap spectrum, exhibiting extensive, confluent purpuric macules and substantial regions of dusky, leaden-hued erythema. The presence of flaccid bullae and noticeable epidermal detachment (positive Nikolsky sign) indicates extensive full-thickness epidermal necrolysis, affecting a larger proportion of the total body surface area compared to Figure S1.

Figure S3	Figure S4	Figure S5

Figure S3 presents a low-magnification overview (X10) of the skin sample, illustrating a notable subepidermal cleft and significant detachment of the necrotic epidermis from the underlying dermis, characteristic of SJS/TEN. In Figure S4, higher magnification (X20) distinctly illustrates the full-thickness epidermal necrolysis, characterized by a detached blister roof composed of eosinophilic, necrotic keratinocytes indicating a complete loss of cellular detail. Ultimately, the high-power magnification in Figure S5 (X40) provides a comprehensive examination of the interface dermatitis, highlighting scattered dyskeratotic (apoptotic) keratinocytes, vacuolar degeneration of the basal layer, and a sparse perivascular lymphocytic infiltrate within the papillary dermis.

Supplementary Table : LTT results and cytokine (granzyme B and granulysin) productions derived from PBMCs of the patients re-exposured with suspicious drugs in vitro assays.

| **Code** | **Stimulants** | **Conc (ug/ml)** | **LTT (SI)** | **Day 3** | | **Day 6** | |
| --- | --- | --- | --- | --- | --- | --- | --- |
|  |  |  |  | **Granzyme B** | **Granulysin** | **Granzyme B** | **Granulysin** |
| SJS01 | w/o |  |  | 1.000 | 1.000 | 1.000 | 1.000 |
| SJS01 | PHA | 1:1,000 | 32.6 | 1488.429 | 6.135 | 752.274 | 167.739 |
| SJS01 | dT | 1:1,000 | 0.8 | 6.652 | 1.146 | 1.857 | 1.477 |
| SJS01 | paracetamol | 1 | 0.9 | 0.806 | 0.106 | 1.336 | 0.736 |
| SJS01 | paracetamol | 10 | 1.1 | 1.100 | 0.519 | 0.361 | 0.496 |
| SJS01 | paracetamol | 100 | 1.8 | 0.902 | 0.630 | 0.826 | 1.170 |
| SJS01 | allopurinol | 1 | 1.3 | 3.150 | 0.377 | 0.883 | 1.238 |
| SJS01 | allopurinol | 10 | 1.6 | 1.100 | 0.114 | 0.743 | 1.087 |
| SJS01 | allopurinol | 100 | 1.6 | 0.714 | 0.308 | 0.431 | 0.709 |
|  |  |  |  |  |  |  |  |
| SJS02 | w/o |  |  | 1.000 | 1.000 | 1.000 | 1.000 |
| SJS02 | PHA | 1:1,000 | 35.1 | 2083.800 | 28.931 | 1649.881 | 109.004 |
| SJS02 | dT | 1:1,000 | 1.2 | 7.874 | 0.972 | 0.894 | 0.210 |
| SJS02 | paracetamol | 1 | 1.4 | 0.875 | 0.683 | 0.229 | 0.132 |
| SJS02 | paracetamol | 10 | 1.8 | 0.585 | 0.475 | 0.164 | 0.054 |
| SJS02 | paracetamol | 100 | 1.7 | NA | 0.878 | NA | 0.240 |
| SJS02 | amikacin | 1 | 1.3 | 2.487 | 1.065 | 4.978 | 5.385 |
| SJS02 | amikacin | 10 | 1.5 | 1.684 | 1.064 | 2.998 | 5.105 |
| SJS02 | amikacin | 100 | 1.4 | 3.270 | 1.198 | 0.598 | 0.554 |
|  |  |  |  |  |  |  |  |
| SJS03 | w/o |  |  | 1.000 | 1.000 | 1.000 | 1.000 |
| SJS03 | PHA | 1:1,000 | 5 | 1447.128 | 73.486 | 2051.357 | 445.392 |
| SJS03 | dT | 1:1,000 | 1.0 | 8.034 | 1.000 | 7.353 | 1.000 |
| SJS03 | paracetamol | 1 | 0.8 | 1.555 | NA | 1.150 | 1.000 |
| SJS03 | paracetamol | 10 | 1.0 | 0.894 | 1.000 | 1.378 | 1.000 |
| SJS03 | paracetamol | 100 | 1.1 | 1.267 | 1.000 | 1.453 | 1.000 |
| SJS03 | Sulfamethoxazole | 50 | 0.9 | 1.027 | 1.000 | 1.303 | 1.000 |
| SJS03 | Sulfamethoxazole | 100 | 0.9 | 0.770 | 1.000 | 1.075 | 1.000 |
| SJS03 | Sulfamethoxazole | 200 | 1.1 | 1.321 | NA | 1.303 | 1.608 |
| SJS03 | Trimethoprim | 10 | 1.0 | 1.501 | 2.829 | 1.453 | 1.000 |
| SJS03 | Trimethoprim | 25 | 1.4 | 1.789 | 11.993 | 0.775 | 1.000 |
| SJS03 | Trimethoprim | 50 | 1.3 | 0.717 | 2.904 | 4.603 | 68.590 |
|  |  |  |  |  |  |  |  |
| SJS04 | w/o |  |  | 1.000 | 1.000 | 1.000 | 1.000 |
| SJS04 | PHA | 1:1,000 | 14.1 | 988.531 | 38.003 | 117.496 | 22.670 |
| SJS04 | dT | 1:1,000 | 0.6 | 3.700 | 0.754 | 0.502 | 0.554 |
| SJS04 | paracetamol | 1 | 1.3 | 0.743 | 0.889 | 0.567 | 0.928 |
| SJS04 | paracetamol | 10 | 1.6 | 0.599 | 0.774 | 0.635 | 0.629 |
| SJS04 | paracetamol | 100 | 2.0 | NA | 1.007 | NA | 0.803 |
| SJS04 | allopurinol | 1 | 1.0 | 1.800 | 1.258 | 1.415 | 1.316 |
| SJS04 | allopurinol | 10 | 1.2 | 0.951 | 0.847 | 0.810 | 0.781 |
| SJS04 | allopurinol | 100 | 1.4 | 0.695 | 0.821 | 0.644 | 0.528 |
|  |  |  |  |  |  |  |  |
| SJS05 | w/o |  |  | 1.000 | 1.000 | 1.000 | 1.000 |
| SJS05 | PHA | 1:1,000 | 121.1 | 61.963 | 7.055 | 160.663 | 36.700 |
| SJS05 | dT | 1:1,000 | 44.2 | 4.023 | 0.977 | 132.221 | 2.653 |
| SJS05 | paracetamol | 1 | 0.7 | 1.159 | 0.943 | NA | NA |
| SJS05 | paracetamol | 10 | 1.1 | 1.311 | 1.031 | NA | NA |
| SJS05 | paracetamol | 100 | 1.0 | NA | 1.170 | NA | NA |
| SJS05 | ethambutol | 10 | 1.1 | 1.442 | 1.122 | 2.733 | 1.681 |
| SJS05 | ethambutol | 50 | 2.6 | 0.751 | 0.769 | 3.982 | 1.823 |
| SJS05 | ethambutol | 100 | 1.2 | 0.528 | 0.532 | 2.191 | 1.355 |
| SJS05 | isoniazid | 1 | 1.0 | 0.680 | 0.830 | 2.259 | 1.709 |
| SJS05 | isoniazid | 10 | 2.3 | 0.608 | 0.692 | 2.643 | 1.914 |
| SJS05 | isoniazid | 100 | 1.2 | 1.573 | 1.044 | 2.065 | 2.111 |
| SJS05 | pyrazinamide | 10 | 4.0 | 0.617 | 0.617 | 1.922 | 1.039 |
| SJS05 | pyrazinamide | 50 | 1.4 | 0.473 | 0.755 | 1.545 | 1.285 |
| SJS05 | pyrazinamide | 100 | 1.8 | 0.517 | 0.719 | 1.168 | 0.922 |
| SJS05 | rifampicin | 10 | 1.5 | 0.396 | 0.423 | 0.949 | 0.655 |
| SJS05 | rifampicin | 50 | 1.9 | 0.867 | 0.646 | 1.164 | 1.015 |
| SJS05 | rifampicin | 100 | 0.9 | 1.194 | 0.984 | 3.441 | 2.404 |
|  |  |  |  |  |  |  |  |
| SJS06 | w/o |  |  | 1.000 | 1.000 | 1.000 | 1.000 |
| SJS06 | PHA | 1:1,000 | 105.9 | 3114.798 | 6.871 | 339.882 | 138.396 |
| SJS06 | dT | 1:1,000 | 19.9 | 1.590 | 1.256 | 2.617 | 0.909 |
| SJS06 | paracetamol | 1 | 1.2 | 1.308 | NA | 0.520 | 0.677 |
| SJS06 | paracetamol | 10 | 0.9 | 1.308 | NA | 0.597 | 0.754 |
| SJS06 | paracetamol | 100 | 1.2 | NA | NA | 0.456 | 0.728 |
| SJS06 | Sulfamethoxazole | 50 | 1.6 | 1.042 | 2.019 | 0.648 | 0.688 |
| SJS06 | Sulfamethoxazole | 100 | 2.5 | 1.888 | 3.729 | 0.712 | 0.697 |
| SJS06 | Sulfamethoxazole | 200 | 4.1 | 2.517 | 6.568 | 0.981 | 0.751 |
|  |  |  |  |  |  |  |  |
| SJS07 | w/o |  |  | 1.000 | 1.000 | 1.000 | 1.000 |
| SJS07 | PHA | 1:1,000 | 30 | 1211.660 | 13.897 | 675.716 | 32.259 |
| SJS07 | dT | 1:1,000 | 6.3 | 0.958 | 0.825 | 3.033 | 0.645 |
| SJS07 | paracetamol | 1 | 1.0 | 1.215 | 1.371 | 0.924 | 0.951 |
| SJS07 | paracetamol | 10 | 1.4 | 1.236 | 1.173 | 1.561 | 0.916 |
| SJS07 | paracetamol | 100 | 0.7 | 1.635 | 1.331 | 0.617 | 0.969 |
| SJS07 | lamotrigine | 1 | 1.2 | 1.346 | 1.238 | 1.089 | 0.944 |
| SJS07 | lamotrigine | 10 | 2 | 1.107 | 1.077 | 1.528 | 0.856 |
| SJS07 | lamotrigine | 100 | 1.6 | 1.236 | 1.055 | 1.257 | 0.898 |
| SJS07 | phenobrabitol | 10 | 1.0 | 1.096 | 1.088 | NA | 1.031 |
| SJS07 | phenobrabitol | 100 | 1.2 | 0.832 | 0.942 | 1.038 | 0.796 |
| SJS07 | phenobrabitol | 200 | 0.7 | 0.780 | 1.125 | 1.751 | 0.871 |
| SJS07 | phenytoin | 10 | 4.8 | 1.434 | 1.085 | 13.226 | 1.101 |
| SJS07 | phenytoin | 50 | 4.4 | 2.068 | 1.089 | 18.749 | 1.143 |
| SJS07 | phenytoin | 100 | 7.4 | 3.213 | 1.423 | 20.625 | 1.749 |
|  |  |  |  |  |  |  |  |
| SJS08 | w/o |  |  | 1.000 | 1.000 | 1.000 | 1.000 |
| SJS08 | PHA | 1:1,000 | 29.7 | 4716.154 | 5.997 | 4114.765 | 23.531 |
| SJS08 | dT | 1:1,000 | 82.7 | 193.136 | 2.400 | 1721.253 | 6.185 |
| SJS08 | paracetamol | 1 | 0.8 | 0.856 | 0.664 | 1.720 | 1.257 |
| SJS08 | paracetamol | 10 | 1.3 | 1.072 | 0.619 | 1.586 | 1.255 |
| SJS08 | paracetamol | 100 | 1.4 | NA | 1.115 | NA | 1.597 |
| SJS08 | amoxicillin | 100 | 1.3 | 0.928 | 0.902 | 1.063 | 1.255 |
| SJS08 | amoxicillin | 200 | 1.7 | 1.218 | 0.693 | 1.192 | 1.196 |
| SJS08 | amoxicillin | 500 | 1.4 | NA | 0.833 | 1.720 | 1.455 |
| SJS08 | clavulanate | 10 | 1.1 | 1.072 | 0.985 | 1.586 | 1.555 |
| SJS08 | clavulanate | 50 | 1.0 | 1.072 | 1.043 | NA | 1.798 |
| SJS08 | clavulanate | 100 | 1.0 | 1.000 | 0.870 | 1.387 | 1.510 |
| SJS08 | ibuprofen | 1 | 0.9 | 0.715 | 0.660 | 0.935 | 0.741 |
| SJS08 | ibuprofen | 10 | 0.9 | 1.292 | 1.037 | 1.991 | 1.097 |
| SJS08 | ibuprofen | 100 | 0.8 | NA | 0.939 | NA | 1.326 |
| SJS08 | lamotrigine | 1 | 2.1 | 2.518 | 1.722 | 1.521 | 1.340 |
| SJS08 | lamotrigine | 10 | 1.0 | 1.146 | 0.824 | 1.063 | 0.979 |
| SJS08 | lamotrigine | 100 | 2.5 | 0.856 | 0.833 | 1.322 | 1.116 |
|  |  |  |  |  |  |  |  |
| SJS09 | w/o |  |  | 1.000 | 1.000 | 1.000 | 1.000 |
| SJS09 | PHA | 1:1,000 | 21.6 | 2989.670 | 108.953 | 263.644 | 151.831 |
| SJS09 | dT | 1:1,000 | 46.3 | 54.032 | 0.814 | 171.639 | 2.472 |
| SJS09 | paracetamol | 1 | 0.6 | NA | NA | 0.840 | 0.842 |
| SJS09 | paracetamol | 10 | 0.5 | 1.667 | NA | 0.761 | 0.956 |
| SJS09 | paracetamol | 100 | 0.4 | 1.000 | NA | 1.065 | 1.411 |
| SJS09 | ceftriazole | 10 | 0.4 | 1.083 | 0.968 | 0.463 | 0.740 |
| SJS09 | ceftriazole | 100 | 0.4 | 0.689 | 0.446 | 0.800 | 0.740 |
| SJS09 | ceftriazole | 200 | 0.4 | 0.920 | 0.724 | 0.482 | 0.796 |
| SJS09 | Methonidazone | 1 | 0.3 | 0.920 | 1.400 | 0.602 | 0.877 |
| SJS09 | Methonidazone | 10 | 0.3 | 1.000 | 0.745 | 0.572 | 0.812 |
| SJS09 | Methonidazone | 100 | 0.5 | 1.958 | 1.825 | 0.721 | 0.954 |
| SJS09 | Tazobactam | 200 | 0.5 | 2.627 | 2.589 | 1.280 | 0.899 |
| SJS09 | Tazobactam | 500 | 0.5 | 2.521 | 3.747 | 0.532 | 0.869 |
| SJS09 | Tazobactam | 1000 | 0.4 | 1.620 | 2.249 | 0.890 | 0.921 |
|  |  |  |  |  |  |  |  |
| SJS10 | w/o |  |  | 1.000 | 1.000 | 1.000 | 1.000 |
| SJS10 | PHA | 1:1,000 | 18.8 | 49.636 | 6.114 | 38.135 | 6.861 |
| SJS10 | dT | 1:1,000 | 5.7 | 1.476 | 1.160 | 3.211 | 1.032 |
| SJS10 | paracetamol | 1 | 1.5 | 0.727 | 1.010 | 0.907 | 0.997 |
| SJS10 | paracetamol | 10 | 1.7 | 0.727 | 1.090 | 0.558 | NA |
| SJS10 | paracetamol | 100 | 1.5 | 0.664 | 1.155 | 1.031 | 0.550 |
| SJS10 | carbamazepine | 10 | 1.3 | 0.623 | 1.108 | 0.876 | 0.982 |
| SJS10 | carbamazepine | 50 | 1.1 | 0.244 | 0.836 | 0.815 | 0.997 |
| SJS10 | carbamazepine | 100 | 1.2 | 0.915 | 1.139 | 1.157 | 0.610 |
|  |  |  |  |  |  |  |  |
| SJS11 | w/o |  |  | 1.000 | 1.000 | 1.000 | 1.000 |
| SJS11 | PHA | 1:1,000 | 5.3 | 810.148 | 5.554 | 121.086 | 7.169 |
| SJS11 | dT | 1:1,000 | 7.6 | 2.346 | 0.916 | 12.119 | 0.835 |
| SJS11 | paracetamol | 1 | 0.8 | NA | NA | 1.680 | 1.252 |
| SJS11 | paracetamol | 10 | 0.6 | 0.666 | 1.007 | 0.923 | 0.956 |
| SJS11 | paracetamol | 100 | 0.8 | 1.462 | 1.036 | 0.853 | 1.176 |
| SJS11 | etoricoxib | 10 | 0.5 | NA | 1.608 | 1.037 | 0.898 |
| SJS11 | etoricoxib | 50 | 0.5 | 1.632 | 1.341 | 0.781 | 0.622 |
| SJS11 | etoricoxib | 100 | 0.6 | 1.462 | 1.577 | 0.644 | 0.727 |
| SJS11 | levofloxacin | 10 | 9.6 | 10.545 | 0.797 | 14.978 | 1.846 |
| SJS11 | levofloxacin | 50 | 5.3 | 13.439 | 1.049 | 16.119 | 3.368 |
| SJS11 | levofloxacin | 100 | 2.0 | 11.603 | 1.007 | 11.703 | 1.497 |
|  |  |  |  |  |  |  |  |
| SJS12 | w/o |  |  | 1.000 | 1.000 | 1.000 | 1.000 |
| SJS12 | PHA | 1:1,000 | 76.5 | 1032.095 | 25.221 | 1.658 | 2.707 |
| SJS12 | dT | 1:1,000 | 1.6 | 1.153 | 0.647 | 1.786 | 2.404 |
| SJS12 | paracetamol | 1 | 1.3 | 1.134 | 0.850 | 1.000 | 1.569 |
| SJS12 | paracetamol | 10 | 1.5 | 0.870 | 0.769 | 1.229 | 1.815 |
| SJS12 | paracetamol | 100 | 1.5 | 1.212 | 1.310 | NA | NA |
| SJS12 | ethambutol | 10 | 2.1 | 1.537 | 0.376 | 2.105 | 0.865 |
| SJS12 | ethambutol | 50 | 2.1 | 0.962 | 0.142 | 1.738 | 0.493 |
| SJS12 | ethambutol | 100 | 1.8 | 0.848 | 0.144 | 1.710 | 0.510 |
| SJS12 | isoniazid | 1 | 1.9 | 0.763 | 0.190 | 1.409 | 1.021 |
| SJS12 | isoniazid | 10 | 2.5 | 1.413 | 1.188 | 1.626 | 2.116 |
| SJS12 | isoniazid | 100 | 2.8 | 1.134 | 1.396 | 1.721 | 4.073 |
| SJS12 | levofloxacin | 10 | 2.4 | 1.872 | 0.499 | 2.510 | 1.293 |
| SJS12 | levofloxacin | 50 | 1.7 | 1.145 | 0.173 | 1.654 | 0.344 |
| SJS12 | levofloxacin | 100 | 2.1 | 0.996 | 0.193 | 1.878 | 0.723 |
| SJS12 | pyrazinamide | 10 | 1.9 | 0.907 | 1.200 | 1.259 | 1.888 |
| SJS12 | pyrazinamide | 50 | 1.9 | 1.252 | 1.000 | 1.114 | 2.030 |
| SJS12 | pyrazinamide | 100 | 1.6 | 1.212 | 1.396 | 2.525 | 5.736 |
| SJS12 | rifampicin | 10 | 1.8 | 1.173 | 1.042 | 0.536 | 0.340 |
| SJS12 | rifampicin | 50 | 1.4 | 0.944 | 0.554 | 1.085 | 1.199 |
| SJS12 | rifampicin | 100 | 1.1 | 0.527 | 0.161 | 2.455 | 3.105 |

LTT results and cytokine (granzyme B and granulysin) productions derived from PBMCs of the patients re-exposured with suspicious drugs in vitro assays.

LTT results were expressed as stimulation index (SI) and their positive results were when SI > 2.0 and expressed with red bold and highlight.

Granzyme B and graulysin productions were expressed as the ratio of each cytokine with each individual's baseline amount of the cytokine (without drug stimulation at corresponding date). Their positive results were expressed when cytokine ratio was more than cut-off points with red bold and highlight. The cut-off points on D3 for GZB and Gr production ratio were 1.835 and 1.665, respectively, as well as the cut-off points on D6 for GZB and Gr production ratio were 1.900 and 1.815, respectively.
